# Supplementary material for: MetaQTL: a package of new computational methods for the meta-analysis of QTL mapping experiments
Source: BMC Bioinformatics. 2007 Feb 8;8:49. doi: 10.1186/1471-2105-8-49 (PMC1808479; doi:10.1186/1471-2105-8-49)
Supplement: Additional File 7 — MetaQTL Package : jar file and tutorial. This Zip archive contains both the MetaQTL JAR file and the files of the tutorial. [file 1471-2105-8-49-S7.zip › org.inra.metaqtl/doc/org/metaqtl/class-use/CMarkerSequence.html]

Uses of Class org.metaqtl.CMarkerSequence


|  |  |  |
| --- | --- | --- |
| **Overview** \| **Package** \| **Class** \| **Use** \| **Tree** \| **Deprecated** \| **Index** \| **Help** | |  |
| PREV   NEXT | **FRAMES**    **NO FRAMES**     **All Classes** |  |


---


## **Uses of Class org.metaqtl.CMarkerSequence**

| Packages that use CMarkerSequence | |
| --- | --- |
| **org.metaqtl** |  |
| **org.metaqtl.algo** |  |

Uses of CMarkerSequence in org.metaqtl

| Fields in org.metaqtl declared as CMarkerSequence | |
| --- | --- |
| `CMarkerSequence` | `ChromCluster.ClusterEdge.cms` |

| Methods in org.metaqtl with parameters of type CMarkerSequence | |
| --- | --- |
| `void` | `ChromCluster.ClusterGraph.addEdge(Chromosome cur1, Chromosome cur2, CMarkerSequence cms)` |

Constructors in org.metaqtl with parameters of type CMarkerSequence

`ChromCluster.ClusterEdge(CMarkerSequence cms,
Chromosome chrom)`

Uses of CMarkerSequence in org.metaqtl.algo

| Methods in org.metaqtl.algo that return CMarkerSequence | |
| --- | --- |
| `static CMarkerSequence` | `CMSAlgorithm.run(Chromosome chrom1, Chromosome chrom2)`             This methods find the common marker sequences between the two given chromosomes. |

---


|  |  |  |
| --- | --- | --- |
| **Overview** \| **Package** \| **Class** \| **Use** \| **Tree** \| **Deprecated** \| **Index** \| **Help** | |  |
| PREV   NEXT | **FRAMES**    **NO FRAMES**     **All Classes** |  |


---
